# Polymorphism of the ITGAM gene (rs7193943) and bioelectric impedance analysis as potential predictors of cachexia in chronic heart failure

**DOI:** 10.1038/s41598-021-99719-6

**Published:** 2021-10-11

**Authors:** Grzegorz Sobieszek, Radosław Mlak, Tomasz Powrózek, Marcin Mazurek, Aneta Skwarek-Dziekanowska, Piotr Terlecki, Teresa Małecka-Massalska

**Affiliations:** 1 Department of Cardiology, 1St Military Clinical Hospital with the Outpatient Clinic, al. Racławickie 23, 20-048 Lublin, Poland; 2 Department of Human Physiology, Medical University of Lublin, Radziwiłłowska 11, 20-080 Lublin, Poland; 3 Department of Vascular Surgery and Angiology, Medical University of Lublin, Lublin, Poland

**Keywords:** Genetic markers, Genotype, Diagnostic markers, Predictive markers, Prognostic markers, Biomarkers, Genetics research, Outcomes research, Nutrition, Prognosis, Weight management, Biomarkers, Cardiology, Risk factors

## Abstract

Cardiac cachexia (CC) is an unfavorable metabolic syndrome leading to exacerbation of chronic heart failure (CHF) and a higher risk of death. The main factor contributing to the development of cachexia is the ongoing inflammatory process mediated by genes (e.g. Integrin Subunit Alpha M—*ITGAM*). The study aimed to assess the relationship between a single nucleotide polymorphism (SNP) -323G > A of the *ITGAM* and the occurrence of nutritional disorders in patients with CHF. 157 CHF patients underwent clinical and nutritional screening. Body composition was evaluated by bioelectrical impedance analysis (BIA). Patients with cachexia were characterized by significantly lower weight, body mass index (BMI), lower fat mass (FM), albumin, and hemoglobin. Lower values of BIA parameters: capacitance of membrane (Cm), phase angle (PA), and impedance ratio (Z200/Z5) were noted in women. Those patients demonstrated significantly higher values of creatinine, c-reactive protein (CRP), N-terminal prohormone of brain natriuretic peptide (NT-proBNP), and pulmonary artery systolic pressure (PASP). A significantly higher risk of cachexia was reported in patients: aged ≥ 74 years (OR 3.55), with renal failure (OR 3.75), New York Heart Association classification (NYHA) III-IV (OR 2.83), with moderate or severe malnutrition according to the score of subjective global assessment (SGA) (OR 19.01) and AA genotype of *ITGAM* gene (OR 2.03). Determination of the -323G > A SNP in the *ITGAM* may prove to be a useful marker (after confirmation in further studies and appropriate validation) in the assessment of the risk of nutritional disorders in patients with CHF.

## Introduction

The incidence of chronic heart failure (CHF) in adults is growing in highly developed countries. The factors associated with the growing number of newly diagnosed cases of CHF are: age (> 70 years) and concomitant diastolic heart failure. As a result of metabolic, anabolic and catabolic disorders associated with CHF, a large number of patients with suspected CHF are diagnosed with left ventricular dysfunction^1, 2^.

Patients with CHF are also often diagnosed with cardiac cachexia (CC). Cachexia is defined as ≥ 5% weight loss over 3–12 months. It is typically accompanied by characteristic symptoms. These symptoms include fatigue, decrease of muscle strength and mass, anorexia, anemia, low albumin level and severe inflammation. The inflammatory process triggers CC pathogenesis and the progression of cachexia. The mortality rate in patients with CC is 20–30% per year. The mechanism of cachexia progression in patients with CHF, in whom the incidence of CC ranges from 8 to 42%, is associated with reduced myocardial perfusion, depletion of high energy stores, cardiomyocyte decline and accumulation of water and lactates in cardiac tissue, accompanied by inflammatory response^3–5^.

The inflammatory process is mediated by genes, including Integrin Subunit Alpha M*—ITGAM* gene (also known as CD11b, Mac-1 integrin alpha chain or complement receptor 3), located on the 16p11.2 chromosome^6^. Its protein product influences interferon gamma (INF-γ) receptor functioning and inflammatory mediator secretion regulation. In the case of low *ITGAM* expression on the surface of antigen presenting cells (APCs), there occurs an increase in the production of interleukin (IL), including IL-6 and IL-17^6, 7^. In patients with systemic lupus erythematosus (SLE), studies have demonstrated the effect of rs114367 single nucleotide polymorphism (SNP) on the level of both the *ITGAM* transcript and monocyte surface protein. The level of mRNA was dependent on the genotype (and the protein amount was proportional to the transcript level). Over twofold decrease in the level of protein was observed in patients with AA genotype (higher risk patients) compared to GG genotype (lower risk group). These differences in the protein expression may be the result of the allele-specific decrease in transcription repression^8^. However, as far as we know, no studies have been performed regarding the correlation between the occurrence of nutritional disorders in patients with CHF and the *ITGAM* gene status. In this study, we have aimed at the assessment of the relationship between *ITGAM* gene regulatory region (−323G > A) SNPs and the incidence of nutritional disorders in cachectic patients with CHF.

## Materials and methods

### Study group

This study included 157 patients (mean age: 69.5 ± 14 years) with the diagnosis of CHF. Enrolled patients were diagnosed and treated at the Clinic of Cardiology and Internal Medicine, Department of Cardiology, Military Hospital in Lublin, Poland in the period of 2013 and 2014. Patients were enrolled in the study consecutively (all newly diagnosed patients with CHF admitted to the cardiology department fulfilling the inclusion and exclusion criteria, respectively were considered). Diagnosis of CHF was made on the latest clinical guidelines issued by European Society of Cardiology (ECS), and the applied criteria involve patients’ clinical screening, echocardiographic assessment (ejection fraction—EF%; left ventricular end-diastolic and end-systolic diameters—LVEDd and LVESd; left atrial diameter—LAD; tricuspid annular piane systolic excursion—TAPSE; right ventricular outflow tract—RVOT) followed by laboratory tests (serum concentration of c-reactive protein—CRP, N-terminal prohormone of brain natriuretic peptide—NT-proBNP, lipid profile, creatinine and hemoglobin concentration)^9^. New York Heart Association (NYHA) functional classification was used to assess extent of the disease by qualifying patients to group I–IV according to the disease severity. The inclusion and exclusion criteria for the study participants were defined, as follows: the inclusion criteria—(a) age > 18 years and polish ethnicity; (b) newly diagnosed CHF; (c) signed informed consent to participate in the study; (d) stable patients without rapid changes in body weight and diuretic treatment for a minimum period of 10 days, and the exclusion criteria—(a) extreme renal failure (according to National Kidney Foundation-Kidney Disease Outcomes Quality Initiative (NKF-K/DOQI) criteria—glomerular filtration rate (eGFR) below 15 mL/min or dialysis); (b) acute coronary syndrome; (c) presence of metallic implants, pacemakers, implanted cardioverter defibrillator or cardiac resynchronization systems; (d) hyper- or hypothyroidism; (e) recent coronary artery bypass grafting (within last 6 months); (f) an active neoplastic process; (g) amputation in the upper or lower limbs. In terms of smoking, the patients were classified as non-smokers, ex-smokers and current smokers. Non-smokers are those who have never smoked or have smoked fewer than 100 cigarettes in their lifetime. Ex-smokers are people who, at the time of the interview, declared that they did not smoke, but had smoked over 100 cigarettes during their lives. The current smoker is what he was smoking at the time of the interview. The patients were followed up for 60 months (5 years) from January 2015 (baseline) until the January 2020. Baseline characteristic of the study group is presented in Table 1. Bioethical Commission in Medical University of Lublin approved the study protocol (no of consent: KE-0254/64/2017).

**Table 1:** Characteristics of the study group.

Variable		Study group (n = 157)
Sex	Men	92 (58.6%)
	Women	65 (41.4%)
NYHA (missing data: n = 2)	I	35 (22.6%)
	II	45 (29.0%)
	III	40 (25.8%)
	IV	35 (22.6%)
SGA (missing data: n = 1)	A	71 (45.5%)
	B	70 (44.9%)
	C	15 (9.6%)
Diabetes mellitus (missing data: n = 1)	Yes	62 (39.7%)
	No	94 (60.3%)
Renal failure (missing data: n = 1)	Yes	59 (37.8%)
	No	97 (62.2%)
Smoking (missing data: n = 1)	Current smoker	67 (42.9%)
	Ex-smoker	34 (21.8%)
	Non-smoker	55 (35.3%)

Continuous variables	Mean ± SD; median (range)	
Age (years)	72.6 ± 13.4; 74.0 (27.0–109.0)	
Weight (kg)	82.0 ± 19.0; 80 (42.0–162.0)	
BMI (kg/m^2^)	29.3 ± 6.1; 29.3 (18.7–53.2)	
Albumin (g/dL)	3.4 ± 0.6; 3.5 (1.4–4.8)	
Triglycerides (mg/dL)	110.5 ± 63.2; 90.4 (33.0–369.0)	
Total cholesterol (mg/dL)	156.1 ± 43.1; 153.5 (60.0–333.0)	
HDL (mg/dL)	49.8 ± 17.5; 49.8 (7.0–110.0)	
LDL (mg/dL)	85.2 ± 34.2; 82.0 (9.8–217.0)	
Creatinine (mg/dL)	1.3 ± 0.5; 1.1 (0.6–3.7)	
Hemoglobin (g/dL)	13.0 ± 2.2; 13.2 (8.8–19.1)	
CRP (mg/L)	16.8 ± 24.2; 6.0 (0.0–16.8)	
Systolic blood pressure (mmHg)	131.4 ± 23.3; 130.0 (80.0–200.0)	
Diastolic blood pressure (mmHg)	75.6 ± 13.6; 75.0 (40.0–130.0)	
EF%	44.0 ± 14.6; 47.7 (0.0–58.6)	
NT-proBNP (pg/mL)	4809.1 ± 7964.4; 2788.0 (138.0–84,919.0)	
LVESd (cm)	4.4 ± 1.0; 4.2 (2.2–7.0)	
LVEDd (cm)	5.6 ± 2.4; 5.4 (3.4–33.0)	
LAD (cm)	4.5 ± 0.6;4.5 (3.0–6.1)	
RVOT (cm)	3.5 ± 0.5; 3.4 (2.3–6.1)	
TAPSE (cm)	2.2 ± 2.5; 1.9 (0.7–25.0)	
PASP (mmHg)	40.9 ± 12.7; 40.0 (3.4–80.0)	

### Nutritional assessment and cachexia detection

Cachexia was diagnosed according to the criteria of Evans et al., as follows: a weight loss ≥ 5% or more in 12 months or less in the presence of underlying illness, plus three of the following criteria: decreased muscle strength, fatigue, anorexia, low fat-free mass index, abnormal biochemistry (increased inflammatory markers: CRP > 5.0 mg/L), anemia (hemoglobin < 12 g/dL) and low concentration of serum albumin (< 3.2 g/dL)^10^. The time points were 15 months at which we measured the parameters. Patients enrolled in this study met the above criteria for cachexia.

Anthropometric measurements (body mass index—BMI, body weight) were taken from all patients and they were also asked to fill in the subjective global assessment (SGA) questionnaire. The bioelectrical impedance analysis (BIA) was used to establish the parameters reflecting the nutritional status and body composition, including: fat mass (FM) and fat-free mass (FFM). The phase angle (PA) (at 50 kHz frequency) and capacitance of membrane (Cm) values were also established using BIA. All study participants had similar conditions of BIA measurements. The measurements were performed in patients lying on the bed in the supine position (patients’ extremities did not come into contact with the torso or each other). The patients rested for at least 5 min before BIA to allow for the equalization of the body fluid level. The body composition parameters were measured using ImpediMed bioimpedance analysis SFB7 BioImp v1.55 device (Pinkenba, QLD, Australia).

Prior to the study, peripheral blood was collected from each study subject in the amount of 5 mL. Before further laboratory analysis , the samples were stored at − 80 °C. The isolation of DNA was conducted using the column method with a dedicated kit according to the manufacturer's recommendations (DNA Blood Mini Kit, Qiagen, Canada). The concentration and quality of the obtained DNA was spectrophotometrically evaluated using NanoDrop Lite Spectrophotometer (Thermo Fisher Scientific, USA). The Real-Time polymerase chain reaction (PCR) technique was used for genotyping reaction on StepOnePlus device (Applied Biosystems, Foster City, CA, USA) in accordance with the manufacturer’s instructions (using Genotyping Master Mix and TaqMan probes specific for *ITGAM* SNP: rs7193943) (Thermo Fisher Scientific, USA). The above methods were also described in detail in our previous studies^11–14^.

### Statistical analysis

Available data were statistically analyzed with the MedCalc 15.8 program (MedCalc Software, Belgium). Results p < 0.05 were considered statistically significant. Comparisons of the values of independent continuous variables were performed using the Mann–Whitney U test (comparison of 2 groups) or ANOVA Kruskal–Wallis test (comparison of more than 2 groups). All selected continuous variables had distribution different than the normal—it was verified by the D`Agostino-Pearson test). To assess the risk cachexia depending on selected demographic, clinical, and genetic factors the odds ratio test (OR) with 95% Confidence Interval (95% CI) (univariate analysis) and logistic regression analysis (multivariate analysis, the model took into account factors significant from the univariate analysis) was used.

### Ethical approval

All subjects gave their informed consent for inclusion before they participated in the study. The study was conducted in accordance with the Declaration of Helsinki, and the protocol was approved of Bioethical Commission in Medical University of Lublin approved the study protocol (no of consent: KE-0254/64/2017).

## Results

### Patients characteristics

The study group consisted of 157 patients diagnosed with CHF. The study group was dominated by men (58.6%), patients with CHF degree II (29%) and III (25.8%) (according to NYHA). The patient median age was 74 years (range: 27–109 years). Patients were mostly well-nourished (SGA A: 45.5%) or in moderate malnutrition (SGA B: 44.9%). Median BMI was 29.3 (range 18.7–53.2). Most of the patients did not have diabetes (39.7%) or renal failure (37.8%). Patients were mostly current smokers (42.9%). Patients classified as non-smokers were in the minority (35.3%). The remaining patients were ex-smokers (21.8%). Detailed data on demographic-clinical and laboratory variables are presented in Table 1.

### Relationships between demographic and clinical factors and ITGAM gene genotypes

In carriers of the GG genotype of the *ITGAM* gene, compared to other variants of studied SNP (AA or GA, respectively), significantly higher values of weight (94 vs 80 or 81 kg), low-density lipoprotein (LDL) (107.7 vs 83.3 or 71.6 mg/dL) and diastolic blood pressure (90 vs 72.5 or 70 mmHg) were found. In addition, a significantly lower incidence of diabetes mellitus was observed in these patients (7% vs. 64.9% or 28.1%). Detailed data of the comparison of selected variables depending on the occurrence of the *ITGAM* genotypes were presented in Table 2.

**Table 2:** Comparison of selected variables depending on the occurrence of the *ITGAM* genotypes.

Variable		*ITGAM* genotypes Median (interquartile range) (missing data: n = 17)			
		AA (1) (n = 72)	GA (2) (n = 60)	GG (3) (n = 8)	*p*
Age (years)		77.0 (39.0.-109.0)	73.0 (27.0–95.0)	67.5 (49.0–82.0)	0.153
Weight (kg)		80.0 (48.0–162.0)	81.0 (42.0–150.0)	94.0 (75.0–130.0)	0.047* 2 vs 3; 1 vs 3
BMI (kg/m^2^)		28.3 (19.5–53.2)	27.7 (18.7–46.9)	33.6 (23.0–37.2)	0.191
FM (kg)		24.9 (2.0–68.6)	22.9. (0.0–65.6)	32.8 (21.9–43.2)	0.339
FFM (kg)		50.8 (0.0–93.3)	52.7 (0.0–85.1)	57.1 (38.1–65.8)	0.090
Albumin (g/dL)		3.4 (1.4–4.6)	3.6 (1.9–4.6)	3.6 (2.4–4.0)	0.311
Triglycerides (mg/dL)		91.8 (40.0–349.0)	84.0 (37.0–369.0)	165.0 (73.9–241.0)	0.065
Total cholesterol (mg/dL)		159.0 (81.0–261.0)	150.0 (60.0–333.0)	181.0 (95.0–235.0)	0.173
HDL (mg/dL)		51.1 (7.0–87.0)	49.0 (9.1–110.0)	49.0 (11.0–65.4)	0.404
LDL (mg/dL)		83.3 (26.0–178.0)	71.6 (9.8–217.0)	107.7 (35.6–149.0)	0.020* 1 vs 2
Creatinine(mg/dL)		1.1 (0.6–2.8)	1.1 (0.7–2.7)	1.2 (0.7–3.7)	0.743
Hemoglobin (g/dL)		13.0(8.0–18.1)	12.9 (8.8–19.1)	13.6 (11.9–16.2)	0.194
CRP (mg/L)		4.8 (0.1–123.0)	8.3 (0.0–117.8)	3.2 (0.6–56.3)	0.256
Systolic blood pressure (mmHg)		130.0 (80.0–200.0)	130.0 (90.0–200.0)	165.0 (115.0–180.0)	0.074
Diastolic blood pressure (mmHg)		72.5 (40.0–100.0)	70.0 (45.0–100.0)	90.0 (70.0–130.0)	0.026* 2 vs 3; 1 vs 3
EF%		41.5 (15.0–65.0)	45.0 (10.0–65.0)	45.0 (20.0–60.0)	0.171
NT-proBNP (pg/mL)		3073.5 (138.0–84,919.0)	2501.0 (285.0–22,534.0)	3039.5 (639.1–18,628.0)	0.830
LVESd (cm)		4.2 (2.7–6.8)	4.0 (2.2–7.0)	4.8 (3.1–7.0)	0.137
LVEDd (cm)		5.3 (3.5–7.5)	5.2 (3.4–33.0)	5.6 (4.8–7.8)	0.135
LAD (cm)		4.5 (3.0–6.1)	4.4 (3.3–5.5)	4.9 (4.0–5.7)	0.149
RVOT (cm)		3.4 (2.3–4.6)	3.3 (2.6–5.0)	3.6 (3.0–6.1)	0.135
TAPSE (cm)		1.9 (0.7–3.0)	1.9 (0.9–9.0)	1.9 (1.4–16.0)	0.800
PASP (mmHg)		40.0 (3.4–80.0)	40.0 (10.0–74.0)	36.0 (22.0–55.0)	0.551
NYHA (missing data: n = 2)	I	14 (46.7%)	14 (46.7%)	2 (6.6%)	0.784
	II	19 (50.0%)	17 (44.7%)	2 (5.3%)	
	III	23 (62.2%)	13 (35.1%)	1 (2.7%)	
	IV	15 (45.5%)	15 (45.5%)	3 (9.0%)	
SGA (missing data: n = 1)	A	27 (43.5%)	29 (46.8%)	6 (9.7%)	0.102
	B	34 (54.0%)	27 (42.9%)	2 (3.1%)	
	C	11 (78.6%)	3 (21.4%)	0 (0.0%)	
Diabetes mellitus (missing data: n = 1)	Yes	37 (64.9%)	16 (28.1%)	4 (7.0%)	0.017*
	No	35 (42.7%)	43 (52.4%)	4 (4.9%)	
Renal failure (missing data: n = 1)	Yes	31 (59.6%)	17 (32.7%)	4 (7.7%)	0.184
	No	41 (47.1%)	42 (48.3%)	4 (4.6%)	
Smoking (missing data: n = 1)	Current smoker	30 (52.6%)	24 (42.1%)	3 (5.3%)	0.870
	Ex-smoker	13 (43.3%)	15 (50%)	2 (6.7%)	
	Non-smoker	29 (55.8%)	20 (38.5%)	3 (5.7%)	
Cm (nF)	Men	1.4 (0.5–3.6)	1.0 (0.4–3.6)	1.1 (0.8–2.0)	0.377
	Women	1.2 (0.0–2.7)	0.9 (0.0–3.6)	0.9 (0.9–0.9)	0.753
Pa (^o^)	Men	3.2 (1.9–9.6)	3.8 (1.5–6.6)	4.8 (4.1–5.1)	0.227
	Women	3.6 (0.0–6.7)	4.0 (0.0–6.6)	3.5 (3.5–3.5)	0.972
Z200/Z5	Men	1.1 (1.1–1.4)	1.2 (1.1–1.3)	1.2 (1.2–1.2)	0.371
	Women	1.2 (1.1–1.3)	1.2 (1.1–1.3)	1.2 (1.1–1.2)	0.671

*Statistically significant results.

### Comparison of selected variables depending on the occurrence of cachexia

Patients with cachexia were characterized by significantly older age (78.5 vs 68.5 years), obviously lower weight and BMI, as well as lower FM (22.9 vs 26.4 kg), albumin (3 vs 3.7 g/dL) and hemoglobin values (12.5 vs 13.8 g/dL), moreover, lower but only in women values of parameters derived from the BIA: Cm (0.7 vs 1.3 nF), PA (2.8 vs 4.2) and impedance ratio—Z200/Z5 (1.1 vs 1.2). On the other hand, in those patients significantly higher values of creatinine (1.2 vs. 1.1 mg/dL), CRP (14.2 vs. 3.6 mg/dL), NT-proBNP (4255.5 vs. 1806 pg/mL) and pulmonary artery systolic pressure—PASP (45 vs. 35 mmHg) were observed (Table 3).

**Table 3:** Comparison of selected variables depending on the occurrence of cachexia.

Variable		Study group (n = 157) Median (interquartile range)		
		Cachectic (n = 74)	Non-cachectic (n = 83)	*p*
Age (years)		78.5 (70.0–86.0)	68.5 (59.0–78.0)	< 0.0001*
Weight (kg)		75.5 (64.8–83.0)	83.0 (75.0–95.0)	0.0001*
BMI (kg/m^2^)		26.9 (24.2–30.1)	30.0 (26.4–33.5)	0.003*
FM (kg)		22.9 (9.4–34.6)	26.4 (19.5–32.9)	0.047*
FFM (kg)		53.6 (39.2–62.8)	50.5 (41.8–59.1)	0.901
Albumin (g/dL)		3.0 (2.7–3.4)	3.7 (3.5–4.0)	< 0.0001*
Triglycerides (mg/dL)		91.7 (69.0–130.0)	90.0 (68.0–143.0)	0.815
Total cholesterol (mg/dL)		148.2 (118.0–176.0)	162.0 (128.0–183.0)	0.258
HDL (mg/dL)		45.0 (37.0–60.0)	49.5 (37.0–63.0)	0.414
LDL (mg/dL)		81.0 (61.8–97.9)	82.8 (61.6–105.4)	0.571
Creatinine(mg/dL)		1.2 (1.0–1.5)	1.1 (0.9–1.3)	0.042*
Hemoglobin (g/dL)		12.5 (11.0–14.0)	13.8 (12.1–14.6)	0.0020*
CRP (mg/L)		14.2 (5.1–33.5)	3.6 (1.6–8.4)	< 0.0001*
Systolic blood pressure (mmHg)		130.0 (110.0–140.0)	130.0 (120.0–150.0)	0.408
Diastolic blood pressure (mmHg)		130.0 (70.0–85.0)	75.0 (70.0–85.0)	0.832
EF%		40.0 (25.0–52.7)	45.0 (30.0–55.0)	0.184
NT-proBNP (pg/mL)		4255.5 (1927.0–8015.0)	1806.0 (1018.0–3333.0)	< 0.0001*
LVESd (cm)		4.0 (3.6–5.2)	4.3 (3.8–5.0)	0.435
LVEDd (cm)		5.3 (4.5–6.2)	5.5 (4.8–6.1)	0.379
LAD (cm)		4.6 (4.0–5.0)	4.5 (4.0–5.0)	0.365
RVOT (cm)		3.5 (3.1–3.8)	3.4 (3.1–3.8)	0.765
TAPSE (cm)		1.8 (1.4–2.0)	1.9 (1.6–2.1)	0.109
PASP (mmHg)		45.0 (35.0–50.5)	35.0 (30.0–45.0)	0.003*
Cm (nF)	Men	1.2 (0.8–1.6)	1.4 (0.7–2.0)	0.687
	Women	0.7 (0.0–1.3)	1.3 (0.9–1.8)	0.009*
PA (°)	Men	3.8 (2.7–4.7)	4.0 (3.1–5.1)	0.169
	Women	2.8 (0.0–3.6)	4.2 (3.6–4.8)	0.004*
Z200/Z5	Men	1.2 (1.1–1.2)	1.2 (1.1–1.2)	0.169
	Women	1.1 (1.1–1.2)	1.2 (1.2–1.2)	0.015*

*Statistically significant results.

### Risk of the cachexia depending on selected demographic, clinical and genetic factors

A significantly higher risk of cachexia was reported in elderly patients (≥ 74: OR 3.55), people with renal failure (OR 3.75), with a higher degree of CHF according to NYHA (III-IV: OR 2.83), with moderate or severe malnutrition according to SGA (B or C: OR 19.01) and AA genotype of *ITGAM* gene (OR 2.03) (Table 4).

**Table 4:** Risk of the cachexia depending on selected demographic, clinical and genetic factors.

Variable	Study group (n = 157)			
	Univariate			Multivariate^#^
	Cachectic (n = 74)	Non-cachectic (n = 83)	OR (95% CI) *p*	OR (95% CI) *p*
**Sex**				
Men	40 (43.5%)	52 (56.5%)	0.70 (0.37–1.33)	0.36 (0.12–1.12)
Women	34 (52.3%)	31 (47.7%)	0.276	0.077
**Age**				
≥ 74	51 (63%)	30 (37%)	3.92 (2.01–7.62)	3.55 (1.63–7.76)
< 74	23 (30.3%)	53 (69.7%)	0.0001*	0.0015*
**Smoking**				
Smoker (current or ex)	46 (45.5%)	55 (54.5%)	0.81 (0.42–1.56)	0.87 (0.44–1.70)
Non-smoker (missing data: n = 1)	28 (50.9%)	27 (49.1%)	0.522	0.454
**Diabetes mellitus**				
Yes	33 (53.2%)	29 (46.8%)	1.47 (0.77–2.80)	1.01 (0.41–2.46)
No (missing data: n = 1)	41 (43.6%)	53 (56.4%)	0.2403	0.9849
**Renal failure**				
Yes	38 (64.4%)	21 (35.6%)	3.07 (1.56–6.01)	3.75 (1.52–9.26)
No (missing data: n = 1)	36 (37.1%)	61 (62.9%)	0.0011*	0.004*
**NYHA**				
II-IV	58 (48.3%)	62 (51.7%)	1.24 (0.58–2.66)	1.37 (0.40–4.54)
I (missing data: n = 2)	15 (42.9%)	20 (57.1%)	0.568	0.615
**NYHA**				
III-IV	46 (61.3%)	29 (38.7%)	3.05 (1.58–5.89)	2.83 (1.04–7.73)
I-II	27 (34.2%)	52 (65.8%)	0.001*	0.042*
**NYHA**				
IV	24 (68.6%%)	11 (31.4%)	3.16 (1.42–7.04)	1.63 (0.47–5.58)
I–III (missing data: n = 2)	49 (40.8%)	71 (59.2%)	0.005*	0.437
**SGA**				
B and C	63 (75%)	21 (25%)	16.36 (7.27–36.81)	19.01 (7.51–48.11)
A (missing data: n = 1)	11 (15.5%)	60 (84.5%)	< 0.0001*	< 0.0001*
**SGA**				
C	14 (93.3%)	1 (6.7%)	18.90 (2.42–147.71)	2.90 (0.31–26.96)
A and B (missing data: n = 1)	60 (42.6%)	81 (57.4%)	0.005*	0.350
**ITGAM**				
AA	42 (58.3%)	30 (41.7%)	2.13 (1.08–4.17)	2.03 (1.01–4.06)
GA and GG (missing data: n = 17)	27 (39.7%)	41 (60.7%)	0.029*	0.048*
**ITGAM**				
GG	2 (25%)	6 (75%)	0.32 (0.06–1.66)	0.35 (0.04–2.63)
AA and GA (missing data: n = 17)	67 (50.8%)	65 (49.2%)	0.176	0.306

*Statistically significant results.

^#^In multivariate analysis, all statistically significant results from univariate analysis were included.

## Discussion

CHF is becoming an ever more prevalent public health issue. The incidence of CHF increases with age, so taking into account the problem of the aging society, the prevalence of CHF is likely to grow in the future largely due to such age-related risk factors as hypertension or chronic inflammation. The inflammatory process taking place in CHF patient is responsible for the nutritional disorders that eventually result in chronic cachexia.

The mechanism of the development of nutritional disorders is significantly affected by the development of inflammation^15–18^. Subsequently, there occurs an increase in the production of the inflammatory cytokines such as tumor necrosis factor alpha (TNF-α), INF-γ, IL-6 and IL-1β. They constitute mediators with confirmed involvement in the mechanisms leading to muscle fiber breakdown in the Ubiquitin–Proteasome Pathway (UPP) through the activation of the muscle ring-finger 1 (*MuRF1*) and muscle atrophy F box (*MAFbx*) ligase genes. It has also been confirmed that the inflammatory process is important in the adipose tissue breakdown. The increase in the number of apoptotic fat cells and decrease in the rate of lipogenesis is associated with increased production of TNF-α. It reduces the glucose and free fatty acid (FFA) transport into cells by interacting with IL-1, which is associated with decreased adipose tissue synthesis. The catabolism of both adipose and muscle tissue also takes place with IL-6 involvement. It may be of great importance for the assessment of the nutritional status and further prognosis to determine the level of this IL^6, 7^.

CC is associated with skeletal myopathy due to genetic changes in a few key regulatory pathways^19^. *ITGAM* gene is of key importance in the activation, migration and adhesion of leukocytes, its main task being the coding of the αMβ2-integrin chain. B-cell signaling and the function of INF-γ receptors, Toll-like receptors (TLR), B-cell receptors, and Fcγ receptors are regulated with the involvement of CD11b. It is an essential molecule in the pathological mechanism of autoimmune diseases, mostly SLE^6^. *ITGAM* may play a role in the production of inflammatory cytokines. It is highly expressed on APCs. If the expression of *ITGAM* on the surface of APCs is low, the production of IL-6 is intensified and, as a result, there occurs T lymphocyte activation and IL-17 production increase^7^. A similar correlation is observed in the case of increased production of other pro-inflammatory factors, namely, IL-1β, TNF α and IFN-β^20^.

We would like to point out that there are no studies directly related to the relationship between *ITGAM* gene status and nutritional disorders in the available literature. The studies that are being available concentrate mainly on *ITGAM* SNPs’ role in rheumatologic conditions—mainly SLE and lupus nephritis (LN)^6, 20, 21^. In our recently published paper, it has been demonstrated that the investigated *ITGAM* SNP (-323G > A) may serve as a useful marker in the assessment of the risk of occurrence of nutritional disorders in head and neck cancer (HNC) patients undergoing radiotherapy (RT)^14^.

In a study conducted on a group of 171 patients with SLE, three SNP variants of the *ITGAM* gene were analyzed: rs1143678 (C > T), rs1143679 (G > A), rs1143683 (C > T) and serum IFN-I level was determined. Each of the analyzed SNPs of the *ITGAM* gene predisposes to a higher risk of developing SLE. It was shown that the tested SNPs increased the serum IFN-I concentration. In a group of 21 people, the GT haplotype common to both rs1143683 (T allele) and rs1143679 (G allele) was confirmed. The GT haplotype was associated with a significantly higher IFN-I concentration in patients diagnosed with SLE (OR 3.57; p = 0.007). The authors also noted that the disease stage (according to Systemic Lupus Erythematosus Disease Activity Index—SLEDAI) and the presence of *ITGAM* SNPs were not associated with significant changes in the level of this cytokine. Increased amounts of produced IFN-I indicate a decreased activity of CD11b and an increased inflammatory reaction in patients with the tested SNP variants of the *ITGAM* gene^22^. In another study in the blood of 30 patients with CHF (NYHA II-IV), an increase in CD11b integrin was observed on the surface of CD45 + leukocytes (p = 0.03) and in the subpopulation of macrophages, monocytes (p = 0.001) and polymorphonuclear cells (p = 0.002). An influence was demonstrated between the advancement of CHF (NYHA) and CD11b expression on the surface of monocytes, macrophages and polymorphonuclear cells (p = 0.001). The increase in the level of this integrin on the surface of these cells is used as a marker of cellular activity^23^. In our study a significantly higher risk of cachexia was reported in elderly patients (≥ 74: OR 3.55), people with renal failure (OR 3.75), with a higher degree of CHF according to NYHA (III-IV: OR 2.83), with moderate or severe malnutrition according to SGA (B or C: OR 19.01) and AA genotype of *ITGAM* gene (OR 2.03). In that aspect AA genotype of *ITGAM* gene may be a useful marker assessing the risk of nutritional disorders in patients with CHF. However, these results cannot be compared with any others as there are none.

It is known that one of the main causes of left ventricular dysfunction and CHF is acute coronary syndrome (AMI) in the form of ST-elevation myocardial infarction (STEMI). In one of the studies, a significantly increased expression of Bone Marrow Stromal Cell Antigen 1—BST1 (sensitivity 77.7778%, specificity 75%) and ITGAM (sensitivity 100%, specificity 75%) was found already in the acute phase of STEMI in which CHF developed at 6 months of follow-up. It was a factor that differentiated patients with post-STEMI CHF from those without CHF. This makes it possible to use new tools for the early prognosis of patients with post-STEMI CHF and the search for genetic potentiality to the evolution of CHF and LV remodeling after STEMI^24^.

The studied SNP’s presence in the regulatory region of the *ITGAM* gene may significantly impact the expression of the gene and, hence, specific protein production. This may lead to the regulation of the production of pro-inflammatory cytokines such as IL-6. Thus, the chronic inflammatory process occurring due to the increased cytokine production, which is characteristic of patients with CHF, may result in the development of nutritional disorders and lead to irreversible conditions like cachexia. Further studies aimed at finding new markers of inflammation involved in the occurrence of malnutrition leading to CC, as well as explaining their mechanisms of action, may facilitate more efficient diagnosis of nutritional disorders, faster implementation of nutritional treatment and the development of new therapies.

Our study has certain limitations, including no assessment of the influence of the diet or of the food intake problems. What is more, we have not investigated the impact of individual genotypes on the expression of the studied gene (and the protein encoded by it). Despite the above limitations, this is, as far as we know, the first study demonstrating that the evaluation of *ITGAM* SNP (-323G > A) may, after confirmation of its value in further studies, constitute a useful marker in assessing the risk of occurrence of nutritional disorders in CHF patients.

## Conclusion

Until now, there are lack of established criteria or clinical guidelines allowing detection and management of CC in CHF patients. However, assessment of genetic markers, such as *ITGAM* (-323G > A) can serve (after appropriate validation) as a putative promising marker for nutritional disorders screening in CHF patients, because of its correlation with patients’ clinical and nutritional features. Prospective clinical utility of the proposed diagnostic marker requires confirmation in large cohort studies.
